# Expression and Characterization of Intein-Cyclized Trimer of *Staphylococcus aureus* Protein A Domain Z

**DOI:** 10.3390/ijms24021281

**Published:** 2023-01-09

**Authors:** Suman Nandy, Vijay M. Maranholkar, Mary Crum, Katherine Wasden, Ujwal Patil, Atul Goyal, Binh Vu, Katerina Kourentzi, William Mo, Amy Henrickson, Borries Demeler, Mehmet Sen, Richard C. Willson

**Affiliations:** 1 William A. Brookshire Department of Chemical and Biomolecular Engineering, University of Houston, Houston, TX 77204, USA; 2 Department of Biology and Biochemistry, University of Houston, Houston, TX 77004, USA; 3 Department of Chemistry and Biochemistry, University of Lethbridge, Lethbridge, AB T1K 3M4, Canada; 4 Department of Chemistry and Biochemistry, University of Montana, Missoula, MT 59812, USA; 5 Escuela de Medicina y Ciencias de Salud, Tecnológico de Monterrey, Monterrey 64849, Nuevo León, Mexico

**Keywords:** protein A, cyclic Z3, SICLOPPS, tandem mass spectrometry, ITC, DSF, SPR

## Abstract

*Staphylococcus aureus* protein A (SpA) is an IgG Fc-binding virulence factor that is widely used in antibody purification and as a scaffold to develop affinity molecules. A cyclized SpA Z domain could offer exopeptidase resistance, reduced chromatographic ligand leaching after single-site endopeptidase cleavage, and enhanced IgG binding properties by preorganization, potentially reducing conformational entropy loss upon binding. In this work, a Z domain trimer (Z3) was cyclized using protein intein splicing. Interactions of cyclic and linear Z3 with human IgG_1_ were characterized by differential scanning fluorimetry (DSF), surface plasmon resonance (SPR), and isothermal titration calorimetry (ITC). DSF showed a 5 ℃ increase in IgG_1_ melting temperature when bound by each Z3 variant. SPR showed the dissociation constants of linear and cyclized Z3 with IgG_1_ to be 2.9 nM and 3.3 nM, respectively. ITC gave association enthalpies for linear and cyclic Z3 with IgG_1_ of −33.0 kcal/mol and −32.7 kcal/mol, and −T∆S of association 21.2 kcal/mol and 21.6 kcal/mol, respectively. The compact cyclic Z3 protein contains 2 functional binding sites and exhibits carboxypeptidase Y-resistance. The results suggest cyclization as a potential approach toward more stable SpA-based affinity ligands, and this analysis may advance our understanding of protein engineering for ligand and drug development.

## 1. Introduction

Protein A of *S. aureus* binds to the Fc portion of human IgG [1,2], helping protect *S. aureus* from opsonophagocytic killing. It also cross-links the antigen-binding (Fab) portions of VH3-type B cell receptors leading to the activation and clonal expansion of B cells and their subsequent apoptotic collapse, a mechanism that suppresses adaptive immune responses during staphylococcal infection [3].

Protein A was first discovered based on the observation that all 500 human sera tested appeared to contain antibodies recognizing *S. aureus* [4]. Further investigation determined that this was not a true immune response but rather was due to a bacterial cell surface protein (protein A) that binds antibodies independent of their antigen specificity [2]. Protein A and its domains have been widely used as model systems for protein folding [5,6] and in immunochemical assays [7]. The functions of protein A as a virulence factor [8,9] and B cell superantigen [10] have been extensively investigated. The Uhlèn group mutagenized the B-domain for greater resistance to hydroxylamine and cyanogen bromide as well as reduced binding to the VH3 Fab region, producing the widely studied ‘Z’ domain [11]. Engineered derivatives of protein A and the Z domain, including forms with increased avidity [12] and resistance to high-pH sanitization, are widely used in the purification of antibodies [13,14]. Display technology has been used to derive Z domain variants binding to non-immunoglobulin targets ‘affibodies’ [15], which are being used for various diagnostic and therapeutic applications [16,17,18].

Stabilizing proteins against thermal stress and proteolytic degradation by topology engineering has been increasingly explored. Tools for enhancing the functional properties of proteins include site-specific incorporation of non-canonical amino acids [19], loop engineering [20], and cyclization [21,22,23]. A potential approach to improving protein stability is the linkage of the N- and C-termini. Side chain cross-linking and backbone cyclization were reported to restrain protein conformation and decrease the entropy of the denatured state, improving stability [24,25]. Cyclization has been achieved by covalent linkage, native chemical ligation (NCL), the actions of inteins, and by enzymatic methods [26,27]. However, low protein yields from NCL, the absence of native cysteine residues in the Z domain to facilitate covalent linkage, energetically unfavorable hydrogen-bonding networks, and interference with appropriate folding [28] remain significant drawbacks of cyclization. Inteins are naturally occurring internal protein domains that autocatalytically remove themselves from the parent protein and re-ligate the flanking regions by spontaneous protein splicing [29]. Split inteins are created by separating inteins’ primary sequence into two fragments at the genetic level. The split polypeptides flank the two termini of the target protein [30]. Upon expression, the intein segments first associate with reconstituting the active intein and then ligating the fused N- and C-extein sequences through protein splicing.

Previous reports demonstrated chemically synthesized cyclic two-helix Z domain protein, high affinity bivalent intein-circularized 2Z protein, and a sortase-mediated cyclized dimer of Z domain affibodies [31,32,33]. In this study, we constructed a cyclic trimer of the Z domain (cyclic Z3) of protein A using gp41-1-based split-intein circular ligation of peptides and proteins (SICLOPPS) [34]. Intein gp41-1 was selected for its superior trans-splicing reaction rate [35,36,37]. The resultant cyclic protein was purified by IgG affinity, confirming the retention of protein A binding functionality. Cyclization was confirmed by matrix-assisted laser desorption/ionization (MALDI), tandem mass spectrometry after trypsin digestion (MS-MS), carboxypeptidase Y (CPDY) digestion, Edman degradation, and analytical ultracentrifugation (AUC). IgG binding of linear Z3 and cyclic Z3 protein was demonstrated using differential scanning fluorimetry (DSF), surface plasmon resonance (SPR), and isothermal titration calorimetry (ITC). DSF showed a similar ca. 5 °C increase in the melting temperature upon IgG binding for both protein variants. ITC demonstrated the similarity of thermodynamics and stoichiometry of association, and SPR showed similar association and dissociation rates. This analysis may advance our understanding of the biophysical effects of topology modification on Z domain-based binding proteins and may also represent an approach to a more stable ligand for use as drugs and in the purification of therapeutic antibodies.

## 2. Results and Discussion

### 2.1. Expression and Purification of Recombinant Linear and Cyclic Z3 Proteins

**Figure 1:**
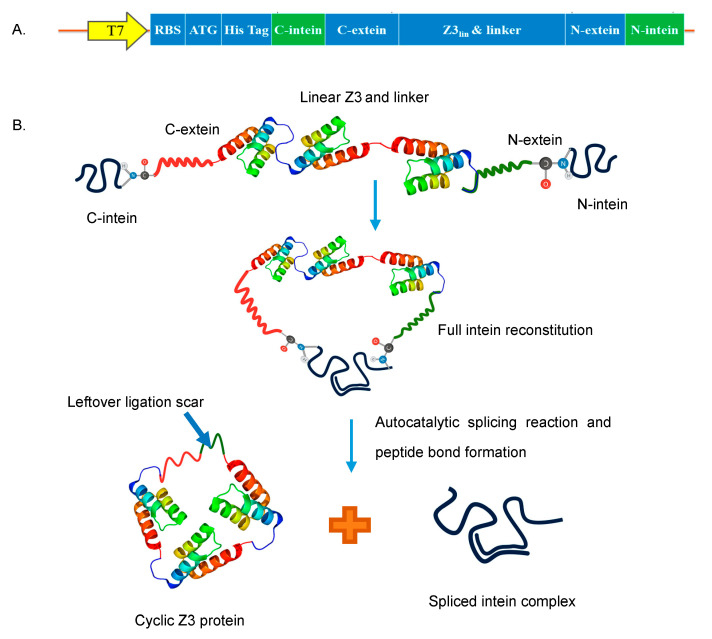
Protein cyclization (**A**) Cyclic Z3 construct map. (**B**) Schematic representation of SICLOPPS process; The two intein fragments interact to form an active intein that autocatalytically splices to cyclize the flanked Z3 protein by forming a peptide bond and releasing the intein complex.

**Figure 2:**
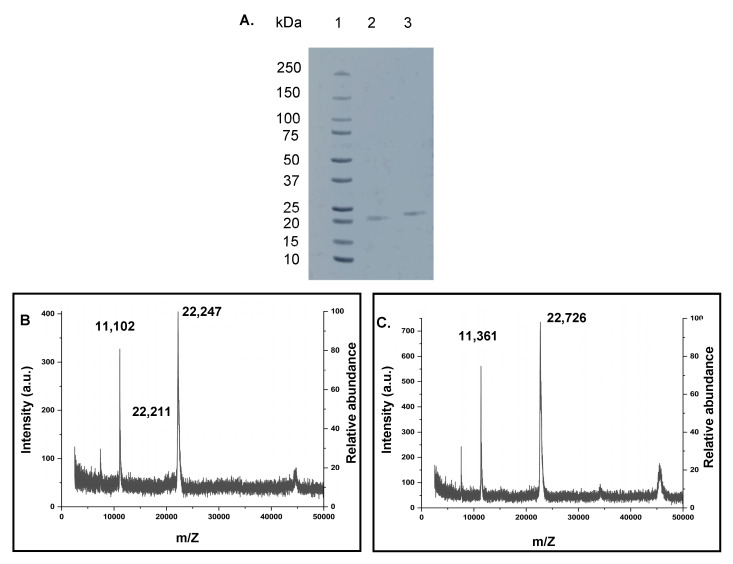
(**A**) Non-reducing SDS-PAGE analysis of cyclization of the Z3 protein. Lane 1: Precision plus protein™ standards (Bio-Rad # 161-0374); lane 2: purified cyclic Z3; lane 3: purified linear Z3. (**B**,**C**) MALDI-ToF mass spectra of purified proteins. (**B**) linear Z3 protein (calculated: 22,394 Da, observed: 22,247 Da and 22,211 Da, additional prominent peak at 11,102 Da attributed to doubly charged ion) (**C**) cyclic Z3 protein (calculated: 22,719 Da, observed: 22,726 Da, additional prominent peak at 11,361 Da attributed to doubly charged ion).

The cyclization of the trimeric Z domain protein was achieved using SICLOPPS. The C-terminal and N-terminal split intein genes flank the two termini of the linear Z3 gene (Figure 1A,B). A hydrophilic α-helical linker (A(EAAAK)_2_A) [38] was inserted between the C terminus of the linear Z3 gene and the N extein, downstream of the Z3 gene to improve folding and avoid destabilizing strain on the native structure. The linear Z3 and cyclic Z3 gene constructs were cloned and transformed into *E. coli* BL-21(DE3) cells and kanamycin-resistant transformants were screened by PCR and agarose gel electrophoresis. Sanger sequencing confirmed the correct sequence of the obtained constructs (pET28a-LinZ3 and pET28a-CycZ3; Supplementary Figure S1). Each recombinant protein was purified using IgG affinity chromatography and immobilized metal affinity chromatography (IMAC), followed by size exclusion chromatography (SEC) as a polishing step (Supplementary Sections S1–S4). Successful IgG affinity purification of the linear and cyclic Z3 confirmed their IgG affinity (Figure S2). Properly spliced and ligated cyclic proteins were purified from non-spliced or prematurely spliced proteins using the IMAC in the flow-through mode and by SEC (Figures S3–S6). As evidenced by non-reducing SDS-PAGE (Figure 2A), the cyclic protein migrated more rapidly on the SDS-PAGE gel than the linear Z3 despite having a 1.5% higher molecular weight, consistent with the more compact nature of the cyclized protein. A similar observation was made with cyclized *Bacillus subtilis* xylanase [29].

### 2.2. Characterization of Recombinant Proteins by Mass Spectrometry Methods

Using the ExPASy Compute pI/MW tool (https://web.expasy.org/compute_pi/, accessed on 20 March 2021), the calculated molecular weight (MW) of linear Z3 protein was 22,394 Da, and that of the cyclic Z3 protein was 22,719 Da. MALDI-TOF gave two singly charged linear Z3 peaks of 22,211 Da and 22,247 Da (Figure 2B). The peak with higher m/Z matches closely with the ExPASy predicted molecular weight, while the lower molecular weight peak in the spectrum might be the product of the co-translational cleavage of N-terminal methionine by methionine aminopeptidase [39]. The MALDI mass of the split-intein-derived ligated cyclic Z3 protein was 22,726 Da, closely matching the calculated mass (+7 Da difference) (Figure 2C). In separate analyses, the purified proteins were subjected to overnight trypsin digestion followed by peptide identification by LC-MS/MS analysis. Mapping analysis confirmed the removal of the terminal methionine residue (Table S1) for the linear Z3 peptide. 

**Figure 3:**
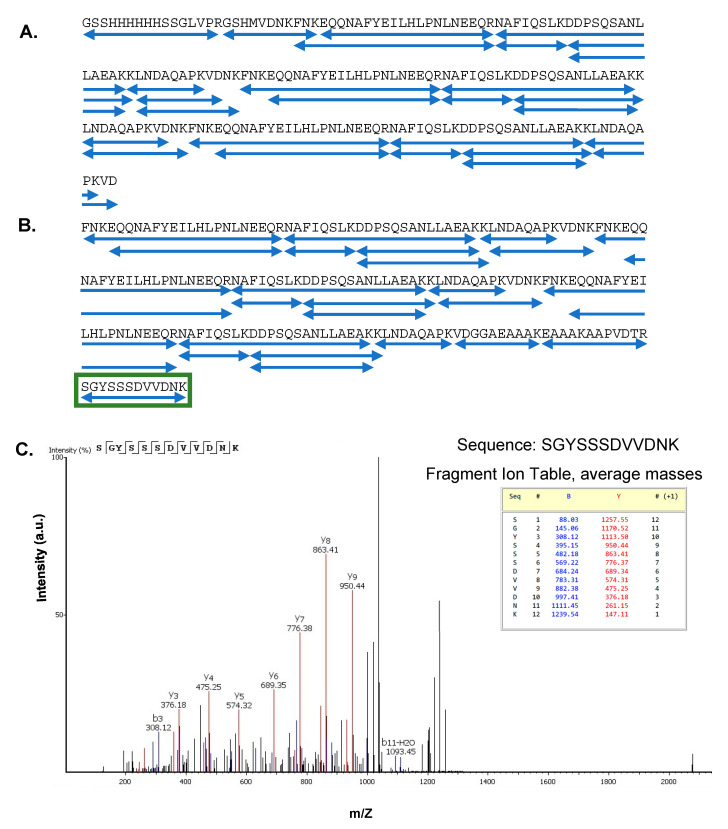
(**A**,**B**) Protein sequence coverage in tryptic MS-MS analyses; (**A**) Linear Z3 protein and (**B**) cyclic Z3 protein. Each blue underline indicates one identified peptide sequence. The green box indicates the peptide sequence connecting the N and C termini of the cyclic Z3 protein. (**C**) Identification of ligation scar sequence in trypsin-digested cyclic Z3. MS/MS spectrum (after trypsin digestion) of the cyclic Z3 protein shows the junction sequence SGYSSSDVVDNK, which is not genetically encoded but is expected in the cyclic protein. The b and y ions corresponding to the ligation scar are shown.

All expected tryptic peptides were observed (Tables S1 and S2), confirming the sequence of each protein (Figure 3A,B). As each protein contains three copies of the Z domain, there is ambiguity in the assignment of each individual peptide molecule to a single location in the overall structure, but no mutations or covalent modifications of the sequence were observed in the tryptic mapping. The overall mass of each protein and, therefore, the number of Z domains was confirmed by other methods. Additionally, the sequence SGYSSSDVVDNK was not encoded in the plasmid DNA but expected in the intein-cyclized protein as the ligation scar sequence between the N and C termini. This sequence was observed in the tryptic peptide data (Table S2, peptide no. 7) from the cyclic Z3 protein (Figure 3C), confirming successful cyclization by SICLOPPS.

### 2.3. Confirmation of Cyclization by Edman Degradation (ED) and Carboxypeptidase Y (CPDY) Digestion

**Figure 4:**
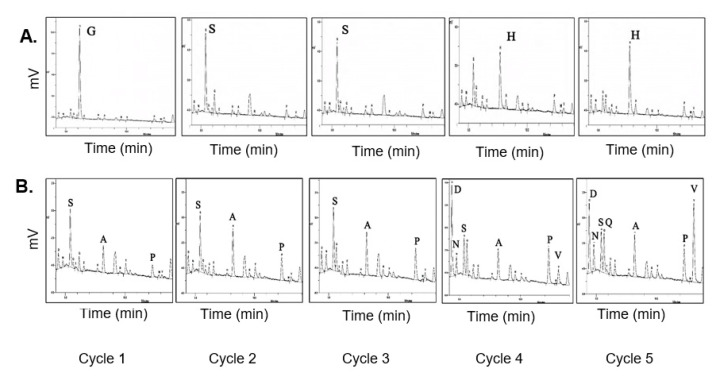
HPLC analysis of phenylthiohydantoin (PTH) amino acids from Edman Degradation. (**A**) N-terminal sequencing of linear Z3 protein. The positions of N-terminal amino acids, assigned for cycle 1 through cycle 5, are labeled. (**B**) N-terminal sequencing of cyclic Z3 protein, the amino acids identified are labeled from the recovered non-cyclized sequences (SSSDV) of splicing intermediates.

Edman N-terminal sequencing was carried out to confirm the absence of a free N-terminus in the cyclic Z3 protein. The Edman degradation-derived N-terminal sequence of the linear Z3 matched with the expected N-terminal sequence (Figure 4A). As expected, Edman degradation of cyclic protein did not give coherent sequencing results; some peaks were observed with the dominant presence of S, A, P, and V amino acid residues (Figure 4B). The expected N-terminal residues of non-cyclized splicing intermediates are SSSDV, and those from the non-spliced parent protein are SSHHH for cycles 1 through 5. The recovered peaks partially matched with the spliced but non-cyclized product of an incomplete SICLOPPS process or protein degradation, as previously discussed [26].

**Figure 5:**
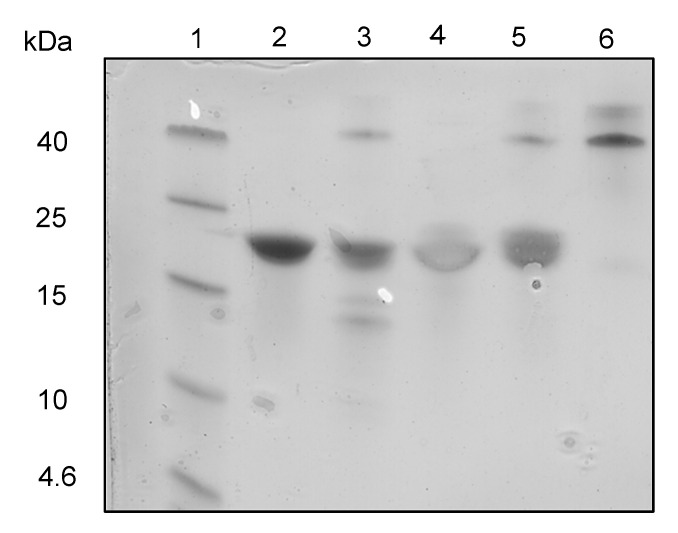
CPDY digestion SDS-PAGE analysis. Lane 1: protein ladder (Spectra Multi Color Low Range; Thermo scientific); lane 2: undigested linear Z3; lane 3: digested linear Z3 (Protein: CPDY mass ratio = 2.5:1); lane 4: undigested cyclic Z3; lane 5: digested cyclic Z3 (Protein: CPDY mass ratio = 2.5:1); lane 6: CPDY enzyme.

Separately, carboxypeptidase Y (CPDY) was used to test for the presence of free C-termini and assess relative resistance to exopeptidase degradation. CPDY is a serine carboxypeptidase with broad amino acid specificity [40]. Two hours of CPDY digestion of the linear Z3 at 37 °C gave lower molecular weight protein fragments (Figure 5), which were not observed with cyclic Z3, suggesting the absence of an accessible carboxyl-terminus and hence resistance to exopeptidase digestion.

### 2.4. Antibody Binding Characterization by Differential Scanning Fluorimetry (DSF)

**Figure 6:**
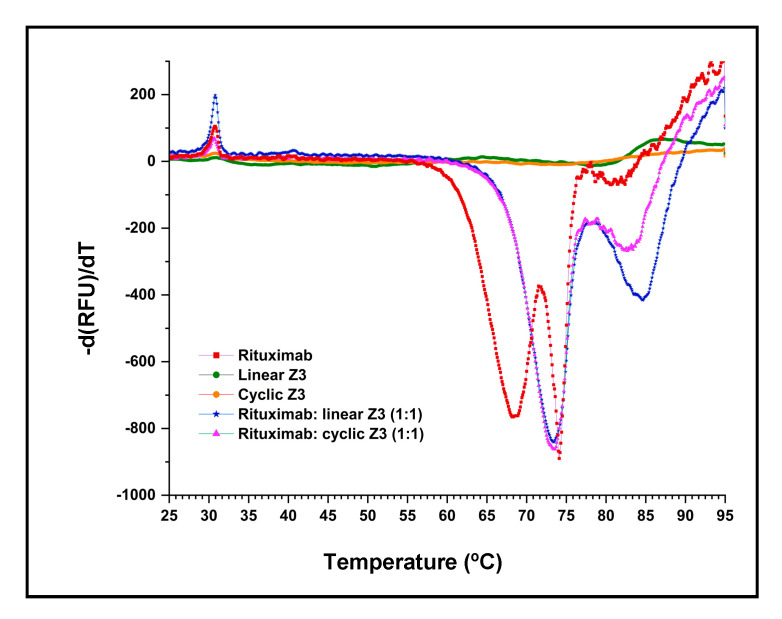
Differential scanning fluorimetry of rituximab-protein interaction in a Bio-Rad CFX 96 real-time PCR instrument. The DSF profiles of equimolar mixtures of rituximab/ Z3 protein variants were compared with profiles of the individual components. The negative derivative of fluorescence is plotted as the temperature was increased from 25 °C to 95 °C.

DSF melting point shift experiments were used to compare the IgG complex stability of the linear and cyclic Z3 proteins. SYPRO™ orange dye binds to the exposed hydrophobic regions of denatured proteins, and so reports temperature-induced denaturation. As shown in Figure 6, the derivative of the fluorescence of Fc and Fab fragments of IgG_1_ shows the denaturation temperature of the Fc and Fab fragments of IgG_1_.

Previous studies have shown that the thermal transitions of the Fc and Fab of IgG_1_ are independent [41]. In our experiments, the DSF profile of rituximab (human monoclonal IgG_1_) showed two major transitions centered at 68.5 ℃ and 74.1 ℃ (Table 1) and one smaller transition at 81.3 ℃, closely matching previous results for human IgG_1_ [42]. No significant change in fluorescence intensity was observed in the linear and cyclic Z3 samples (Figure 6) over the temperature range of interest. The high denaturation temperature of the Z domain was previously reported (melting temperature >90 °C, as determined by DSF and circular dichroism) [42].

As expected, binding protein A variants to rituximab resulted in an increase in both transition temperatures. Addition of either linear or cyclic Z3 increased the lower transition temperature by 4.9 °C, suggesting that each Z3 variant interacts similarly with Fc (Table 1). The transition observed at 81.3 °C with pure rituximab (ascribed to the Fab and secondary Fc interactions [42] shifted upward by 3.3 °C and 1.5 °C for rituximab/protein complexes with linear and cyclic Z3, respectively. In addition, thermodynamic parameters of unfolding at 25 °C were calculated for the rituximab Fc transition near 68.5 °C (Table 1), assuming a two-state reversible protein folding model with no stable intermediate [43]. ∆_u_G^0^ and ∆_u_H^0^ of rituximab/Z3 protein variants in a 1:1 molar mixture were similarly higher than those of rituximab (Table 1). However, the addition of neither protein A form produced a significant change in the ∆_u_S^0^. The increase in T_m_, ∆_u_G^0,^ and ∆_u_H^0^ implies similar molecular interactions between the Z3 protein variants and rituximab.

### 2.5. Binding Kinetics by Surface Plasmon Resonance (SPR)

**Figure 7:**
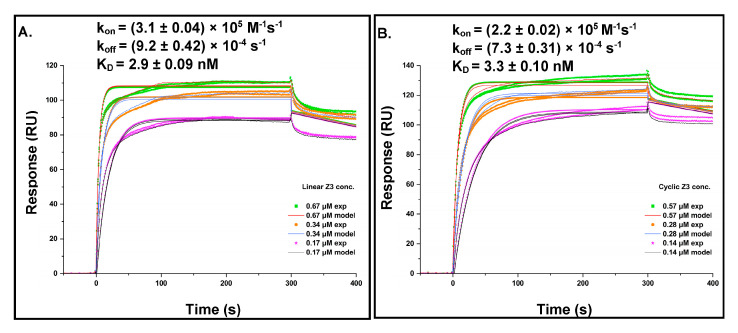
Kinetics of Z3 proteins interaction with rituximab (**A**) Linear Z3 at 0.67, 0.34, and 0.17 μM and (**B**) cyclic Z3 at 0.57, 0.28, and 0.14 μM were bound to immobilized rituximab. Rate and equilibrium constants were determined from direct curve fitting of the sensorgrams. The values represent the mean ± standard error calculated from the BIAevaluation software.

Dose–response sensorgrams were obtained for the binding of linear and cyclic Z3 to rituximab immobilized on a CM5 dextran chip. Kinetic parameters were calculated using the BIAevaluation software (Version 2.0.1) with a 1:1 Langmuir binding model (Figure 7A,B). The model-derived association rate constants (k_on_) for linear and cyclic Z3 were (3.1 ± 0.04) × 10^5^ M^−1^s^−1^ and (2.2 ± 0.02) × 10^5^ M^−1^s^−1^, respectively. The dissociation rate constants (k_off_) were (9.2 ± 0.42) × 10^−4^ s^−1^ and (7.3 ± 0.31) × 10^−4^ s^−1^, respectively, for linear and cyclic Z3. The dissociation equilibrium constants, K_D_, calculated as k_off_/k_on_, was 2.9 ± 0.09 nM for linear Z3 and 3.3 ± 0.10 nM for cyclic Z3. The kinetics-derived k_on_, k_off_, and dissociation constant for linear Z3 are in reasonable agreement with literature values determined for linear ZZ independently by flow cytometry, ELISA, and SPR [32,44,45]. Although the k_on_ and k_off_ values for cyclic Z3 were slightly (29% and 21%, respectively) lower than those for the linear Z3, the K_D_ was nearly identical (Figure 7A,B). The similar affinities of the two protein variants for rituximab may be attributed to their functionally similar binding surfaces and confirm that cyclization does not significantly interfere with binding either by steric hindrance or by local denaturation.

### 2.6. Conformational Analysis by Analytical Ultracentrifugation (AUC)

**Figure 8:**
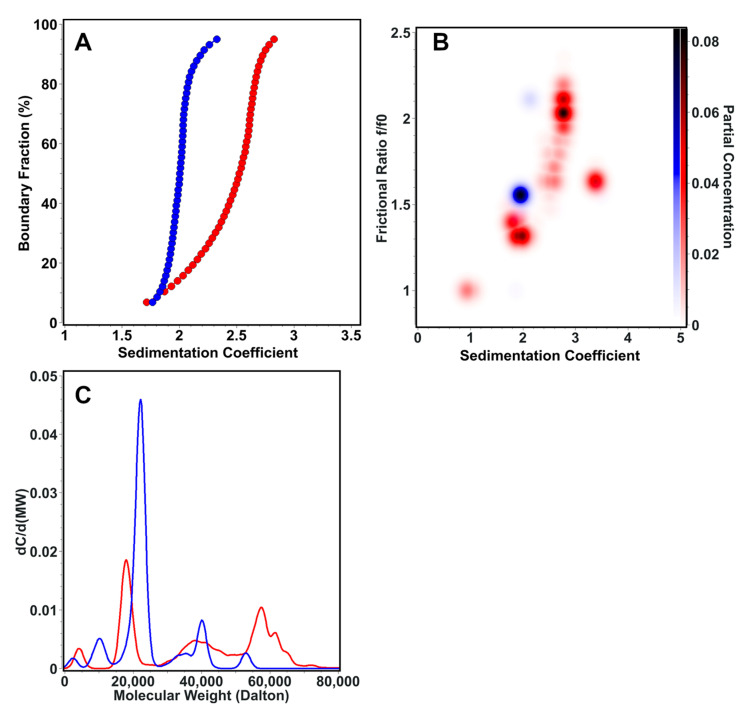
AUC sedimentation velocity experiment results for the linear (red) and cyclic (blue) forms of the Z3 molecule. (**A**) Diffusion-corrected van Holde—Weischet sedimentation coefficient profiles, showing a higher degree of heterogeneity for the linear form. (**B**) Global Monte Carlo analysis of the frictional ratio of linear and cyclic Z3, showing also greater heterogeneity in the frictional ratio for linear Z3. (**C**) Molar mass overlay of linear and cyclic Z3.

AUC sedimentation velocity experiments were used to characterize the sedimentation and transport properties of the linear and cyclic Z3 molecule. The linear form (S = 1.7–2.9) has higher sedimentation heterogeneity than the cyclic form (S = 1.7–2.2) (Figure 8A). The frictional ratio of the cyclic form appeared primarily as a single, homogeneous species at f/f0 = 1.5 and S = 2.0, While linear Z3 was more heterogeneous with a frictional ratio f/f0 = 1.3–2.2 and S = 1.7–2.9 (Figure 8B). Molar mass transformations of the sedimentation and diffusion coefficients, assuming a constant partial specific volume, suggest that the linear form is more prone to forming oligomeric structures as large as trimers, while the cyclic form remains mainly monomeric (Figure 8C). These results suggest that the linear form is more prone to oligomerization. In contrast, the cyclic form is more compact, less heterogeneous, and has less conformational flexibility than the linear Z3, a finding that is consistent with previous literature on cyclic proteins [46,47].

### 2.7. Binding Thermodynamics by Isothermal Titration Calorimetry (ITC)

**Figure 9:**
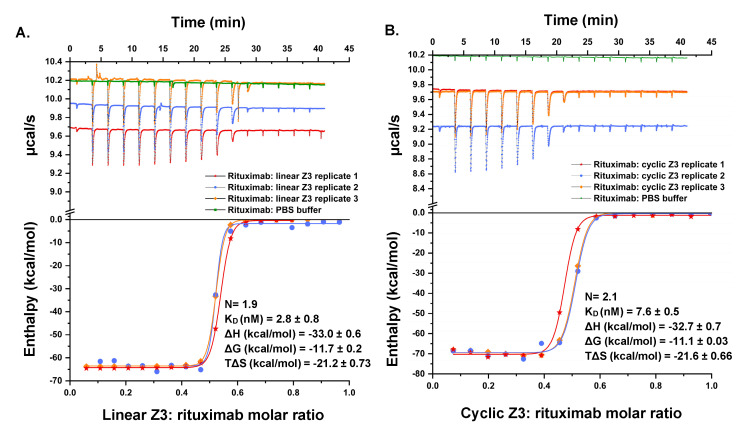
Titration calorimetric characterization of the interaction between rituximab and Z3 protein variants. (Top) Raw data of the sequential titration of (**A**) 40.3 μM linear Z3 into 8.0 μM rituximab in the sample cell and (**B**) 48.1 μM cyclic Z3 into 7.6 μM rituximab in the sample cell. (Bottom) B integrated heat of titration as a function of the molar ratio of Z3: rituximab. The standard error was calculated from three independent measurements.

ITC was used to analyze the energetics of association of Z3 proteins with rituximab (Figure 9). The binding of linear Z3 to rituximab was enthalpically driven (∆H = −33.0 ± 0.6 kcal/mol), with an unfavorable entropic contribution at 25 °C (T∆S = −21.2 ± 0.73 kcal/mol) and an overall Gibbs Free energy of binding, ∆G of −11.7 ± 0.2 kcal/mol (K_D_ = 2.8 ±  0.8 nM) and binding stoichiometry of 1.9 IgG_1_ molecules per linear Z3. The energetics of binding of cyclic Z3 was very similar with ∆H = −32.7 ± 0.7 kcal/mol, T∆S = −21.6 ±  0.66 kcal/mol, ∆G = −11.1 ±  0.03 kcal/mol, K_D_ = 7.6 nM and stoichiometry of 2.1 IgG_1_ molecules binding per cyclic Z3 molecule. A study of Z2 domain protein association with human IgG showed energetics similar to those observed here, with a favorable binding enthalpy (ΔH = −34.5 kcal/mol) and unfavorable binding entropy (TΔS = −23.9 kcal/mol) [48]. Additionally, the thermodynamic properties obtained in this study are in accord with previously reported studies on protein A-IgG interaction [48,49]. Both linear and cyclic Z3 contain three identical IgG_1_ binding monomers, but the ITC data for each revealed functional stoichiometry of ~2 IgG_1_ per Z3 protein variants, presumably because of steric exclusion [48]. These results are in agreement with the previously reported solution phase stoichiometry of antibodies and Fc-fusion proteins with protein A (GE Healthcare and Calbiochem), measured using ITC and SEC [50,51]. Furthermore, while we had anticipated a potential reduction of conformational entropy loss upon binding by cyclization, we observed no significant difference in binding energetics, suggesting domain independence.

## 3. Materials and Methods

### 3.1. Materials

Synthetic nucleic acids were obtained from Genewiz (South Plainfield, NJ, USA). Bovine serum albumin (BSA), chicken egg-white lysozyme, Benzonase^®^ Endonuclease, LB broth (Miller), isopropyl ß -D-1-thiogalactopyranoside (IPTG), Tris, imidazole, Amicon centrifugal filters, tryptone enzymatic digest from casein, sodium chloride, Triton X-100, kanamycin, carboxypeptidase Y (CPDY) and spermidine tetrahydrochloride were purchased from Sigma (St. Louis, MO, USA). Pierce™ protease inhibitor mini tablets EDTA-free, SYBR Green I, Pierce™ BCA protein assay kit, and SYPRO™ orange protein gel stain were purchased from Thermo Fisher Scientific (Waltham, MA, USA). Other reagents used include glacial acetic acid, glycerol, sodium hydroxide, hydrochloric acid, anhydrous ethanol (VWR, Radnor, PA, USA), and phosphate-buffered saline (PBS) tablets Takara Bio (San Jose, CA, USA). Buffers were prepared with deionized water (Millipore Milli-Q, Burlington; MA, USA) and filtered with sterile polystyrene filters (Corning, New York, NY, USA). Gibson Assembly^®^ cloning master mix (GA), restriction enzymes, and Q5^®^ High-Fidelity Polymerase 2X Master Mix were purchased from Promega (Madison, WI, USA) or New England Biolabs (Ipswich, MA, USA). QIAprep Spin Miniprep, QIAquick Gel Extraction Kit, QIAquick polymerase chain reaction (PCR) cleanup, and QIAprep plasmid miniprep kits were purchased from Qiagen (Germantown, MD, USA). IgG Sepharose 6 Fast Flow, Ni Sepharose 6 Fast Flow adsorbents, XK 16/200 columns, and Superdex-200 10/300 GL SEC column were purchased from Cytiva (Marlborough, MA, USA). Transformation-competent *E. coli* BL-21(DE3) cells and pET plasmids were obtained from Invitrogen (Waltham, MA, USA). Rituximab was purchased from Zydus (Ahmedabad, Gujarat, India).

### 3.2. Circularized and Linear Z3 Protein Plasmid Construction

A synthetic gene sequence encoding a single copy of the Z domain (RCSB PDB—2SPZ) flanked by gp41-1 inteins (C terminal intein/extein and N-terminal intein/extein [37]) was purchased from Genewiz. PCR amplification was performed using high-fidelity DNA polymerase (Q5^®^ High-Fidelity 2X Master Mix). The gene fragment was amplified with the forward primer NdeI-Zcyc-F (5′ CCGCGCGGCAGCCATATGATGTTGAAGAAGATC 3′, encoding the NdeI restriction site) and the reverse primer XhoI-Zcyc-R (5′ GTGGTGGTGGTGGTGCTCGAGTTACTTGTC 3′), encoding the stop codon and the XhoI restriction site), and cloned into a NdeI/XhoI digested pET28a vector using 2X GA reaction mix to produce pET28a-Z1_cyc_. Afterward, the SalI restriction site encoded downstream of the Z domain was used to produce gene-coding cyclic Z domain multimers. A linear double-stranded 5′ phosphorylated Z domain gene with the N-terminal intein/extein gene was obtained for Z domain multimerization purposes. First, the plasmid pET28a-Z1_cyc_ was digested with SalI and XhoI. The linear double-stranded 5′ phosphorylated gene was then ligated to linearized pET28a-Z1_cyc_. The resultant cyclic Z protein-coding gene was amplified using primers NdeI-Zcyc-F and XhoI-Zcyc-R, analyzed by gel electrophoresis, and visualized by SYBR Green staining. Correct-sized fragments were excised from the gel, purified using a QIAquick gel extraction kit, and cloned into the pET28a vector using a 2X Gibson assembly (GA)reaction mix to produce the plasmid pET28a-Z2_cyc_. Briefly, a purified gene fragment (30 ng/μL) was incubated with NdeI/XhoI digested pET28a (50 ng/μL) and 2X GA reaction mix at 50 °C for 2 h. The same procedure was repeated to produce the cyclic Z3 protein-expressing plasmid pET28a-Z3cyc. Separately, linear Z3 protein-expressing plasmid, pET28a-Z3lin was produced according to our previously reported study [52].

### 3.3. Expression and Purification of Linear Z3 and Cyclic Z3 Proteins

A 5 mL aliquot of freshly transformed *E. coli* BL-21(DE3) cells in LB medium was incubated for 4–5 h at 37 °C and then used to innoculate to 2.8 L baffled Fernbach flasks containing 500 mL Super Broth (32 g tryptone, 20 g yeast extract, 5 g NaCl, 5 mL 1 N NaOH) with 50 μg/mL kanamycin. When the cultures reached an OD600 of 0.6, protein expression was induced with 1 mM IPTG and grown for an additional 5 h. Bacterial cells were then harvested in an Avanti J-E High-Speed Centrifuge (Beckman Coulter, CA, USA) at 6000× *g* for 20 min and stored at −80 °C until further use.

Bacterial pellet was mixed with half of its volume of a lysis buffer (20 mM Tris-HCl buffer, pH 8.0, 100 μg/mL lysozyme, 0.1% Triton X-100, 10 mM spermidine tetrahydrochloride, and 10% glycerol). The mixture was incubated at 4 ℃ for 1 h, and the cells were lysed using a Biologics 150VT Ultrasonic Homogenizer at 40 kHz for three 15 s cycles. The cell lysate was centrifuged at 14,500× *g* for 20 min using an Eppendorf Centrifuge 5424 R. The supernatant was filtered using a 0.22 μm syringe filter, loaded on an IgG Sepharose FF column, and eluted using 0.1 M acetic acid, pH 3.5. The eluate was pH adjusted to pH 7.4 using 2 M Tris solution and loaded on a Superdex-200 SEC column to remove the aggregates and fragments.

For cyclic Z3 purification, the IgG Sepharose affinity-capture step was carried out as described in the previous paragraph. The neutralized eluate was subjected to IMAC using Ni Sepharose 6 Fast Flow adsorbents in flow-through mode. Furthermore, the Superdex-200 SEC column was used to remove aggregates and fragments. (See Supplementary information Section S4).

### 3.4. SDS-PAGE

SDS-PAGE was performed using 4–20% Mini-PROTEAN^®^ TGX™ Precast Gels (Bio-Rad, CA, USA), according to the manufacturer’s recommendation. All samples for electrophoresis were mixed with 2X Laemmli SDS-PAGE gel loading buffer in 1:1 (*v/v*) and loaded on the gel. Finally, the gel was stained with 0.1% (*w/v*) Coomassie blue R-250, 40% (*v/v*) methanol, and 10% (*v/v*) acetic acid.

### 3.5. Matrix-Assisted Laser Desorption/Ionization (MALDI) Mass Spectrometry

The purified proteins were characterized by MALDI-ToF-MS (Voyager STR-DE, Applied Biosystems, Waltham, MA, USA) analysis at the Tufts University Core Facility (Boston, MA, USA). For mass spectrometry, protein samples were buffer exchanged in Tris–HCl buffer (0.02 M, pH 7.4) on a Superdex-200 SEC column, concentrated using Amicon centrifugal filters (2 mg/mL; quantified with the Pierce™ BCA protein assay kit), and submitted for analysis frozen on dry ice. Sinapinic acid was used as a MALDI matrix, with a 0.5 μL spot of sample and 0.5 μL of matrix on top of it on the stainless-steel sample plate. The mixture was air-dried and loaded into the instrument for reading in linear mode.

### 3.6. Tryptic Digestion MS-MS

The protein samples (50 μL of 1 mg/mL) were heat-treated at 95 ℃ for 5 min. Then, Thermo Pierce™ Trypsin Protease, MS Grade (catalog number: 90058), was added in a proportion of 1:40 (Z3/trypsin, *w/w*), and the mixture was incubated overnight at 37 ℃ and 500 rpm on a turbo thermal shaker. The samples were cleaned up using Thermo Scientific Pierce^TM^ C18 Tips on a 100 µL bed as per the manufacturer’s recommendations [53]. Trypsin-digested samples were analyzed on a mass spectrometer (timsTOF Pro) MS-MS analyzer at the Mass Spectrometry Laboratory, University of Houston, Houston, Texas, USA.

### 3.7. Carboxypeptidase Y (CPDY) Digestion

Cyclic and linear Z3 protein samples were buffer exchanged into 50 mM, pH 6.5 MES (2-(N-morpholino) ethanesulfonic acid) buffer. Lyophilized CPDY powder was dissolved in 50 mM, pH 6.5 MES buffer. Linear and cyclic Z3 protein samples (0.2 mg/mL of 0.1 mL) were mixed with CPDY enzyme in a mass ratio of 2.5:1 (*w/w*) and incubated at 37 °C for 2 h (the reaction conditions were obtained from the literature [54]). The resultant post-reaction samples were analyzed on SDS-PAGE as described above.

### 3.8. Edman Degradation

N-terminal amino acid sequencing using Edman degradation was conducted at Tufts University Medical School (Boston, MA, USA). The protein samples were spotted onto polyvinylidene fluoride (PVDF) using a ProSorb cartridge from Life Technologies, and automated Edman degradation was performed on an Applied Biosystems Procise 494 HT instrument. Data analysis was carried out using the instrument’s proprietary software.

### 3.9. Differential Scanning Fluorimetry (DSF)

DSF assays were used to characterize the thermal stability of proteins and of complexes of linear and cyclic Z3 with IgG_1_ [55]. Proteins (32 μM) were mixed with 10X SYPRO™ orange dye (stock concentration: 5000X in DMSO) in 20 mM Tris, pH 7.4. The total reaction volume per well in the PCR plate was 20 μL. The fluorescence was measured at 15 s interval with a temperature gradient of 0.6 °C/min from 15 °C to 95 °C in a Bio-Rad CFX96 real-time PCR instrument [56].

### 3.10. Surface Plasmon Resonance (SPR)

SPR experiments were performed using a Biacore X100 system (Cytiva, Marlborough, MA, USA). Rituximab was covalently immobilized in flow cell 2 of a carboxy methylated (dextran) CM5 sensor chip, and flow cell 1 was used as a reference channel. First, the carboxymethyl groups of the chip were activated with 70 μL 0.05 M N-hydroxysuccinimide (NHS) and 0.2 M 1-ethyl-3-(3-dimethylamino-propyl)-carbodiimide hydrochloride (EDC) solution. Multiple injections of 70 μL rituximab (50 μg/mL) were made to promote the coupling reaction by washing with 10 mM HEPES, 150 mM NaCl, 3 mM EDTA, 0.005% Tween-20, pH 7.4 (HBS-EP+); (previously 0.22 μm filtered and sonicated) between injections. Upon baseline stabilization, the uncoupled activated carboxymethyl groups were blocked with 70 μL 1 M ethanolamine-HCl, pH 8.5. Following the same procedure, the reference channel (flow cell 1) was blocked in parallel with 1 M ethanolamine (10 μL/min flow rate was used in all steps of chip preparation). For the binding study, serial dilutions of purified buffer-exchanged Z3 samples covering a range of 100 nM–10 μM were freshly prepared in running buffer HBS-EP+; 75 μL (300 s) were injected for each dilution for the association. Dissociation was performed at 15 μL/min over a 120 s interval with the HBS-EP+ buffer. The surface was regenerated between samples by injecting 45 μL (90 s) of 100 mM sodium citrate, 500 mM NaCl, pH 3.5. All experiments were carried out in triplicate, and results were analyzed using the Biacore X100 BIAevaluation software version 2.0.1 (Cytiva, Marlborough, MA, USA).All experiments were carried out in triplicate, and results were analyzed using the Biacore X100 BIAevaluation software, version 2.0.1 (Cytiva, Marlborough, MA, USA).

### 3.11. Analytical Ultracentrifugation (AUC)

AUC experiments were performed to compare the composition and conformation of linear and cyclic Z3 molecules. All samples were measured on an Optima AUC (Beckman Coulter, Indianapolis, IN, USA) at the Canadian Center of Hydrodynamics, University of Lethbridge, AB, Canada. Sedimentation velocity (SV) data were analyzed using UltraScan-III, version 6570 [57]. AUC experiments were performed in PBS (Dulbecco). SV experiments were measured twice at two speeds, first at 40,000 rpm for 16 h and then at 60,000 rpm for 7 h. The temperature in both experiments was maintained at 20 °C, and samples were monitored in intensity mode at a wavelength of 220 nm. The data from the two different speeds were fitted globally using the two-dimensional spectrum analysis [58] in conjunction with Monte Carlo analysis [59] to optimize the frictional information [60]. SV experiments were processed as described earlier [61]. Diffusion-corrected sedimentation profiles were determined with the enhanced van Holde—Weischet analysis [62]. For calculating the molar mass and frictional ratios, a partial specific volume of 0.7206 mL/g was assumed, as calculated by UltraScan based on the sequence information. Furthermore, the presence of contaminants or aggregates in the samples was resolved to identify the hydrodynamics of the species of interest.

### 3.12. Isothermal Titration Calorimetry (ITC)

ITC studies were performed using a MicroCal PEAQ-ITC instrument (Malvern, UK). Before performing any experiment, a system suitability test was performed as per the manufacturer’s recommendation (Figures S7 and S8 and Table S3). The samples were buffer-exchanged into PBS, pH 7.4, using a Cytiva Superdex-200 SEC column and degassed by incubating in a vacuum for 20 min. The concentration of the analytes was optimized to reach saturation at least four injections before titration ended. A stirring speed of 750 rpm, temperature 25 ℃, reference power 10 μcal/s, high feedback, and an initial delay of 60 s was used for all experiments. The first priming injection was 0.4 μL, and the rest were 2 μL with 150 s intervals between injections. All calorimetric measurements were performed in triplicate. The data were analyzed using MicroCal PEAQ-ITC analysis software (Version 1.1.0.1262).

### 3.13. Accession Codes

UniProt ID of *Staphylococcus aureus* protein A is P38507·SPA_STAAU, rituximab is P0DOX5·IGG1_HUMAN, and carboxypeptidase Y is P00729·CBPY_YEAST. Protein Data Bank ID for staphylococcal protein A Z domain is 2SPZ.

## 4. Conclusions

We describe the development of a cyclic trimer of protein A Z domain using gp41-1 intein-based protein splicing and comparison of the biophysical characteristics of the cyclic and linear molecules. Overall biophysical characterization showed functional near-equivalence between cyclic and linear proteins. Cyclization appears to reduce conformational heterogeneity and should increase exopeptidase and ligand leaching resistance during processes such as the purification of monoclonal antibodies. Further studies will be required to demonstrate the potential advantages of the cyclic ligand. Nevertheless, structural and biophysical studies of engineered IgG-binding domains and their complexes are of interest for developing Z domain-based affinity tools as well as for further understanding the mechanisms of biomolecular recognition.

## Figures and Tables

**Table 1:** Melting temperatures (T_m_) and thermodynamic parameters of unfolding (Gibbs free energy, enthalpy, and entropy change) of rituximab and an equimolar mixture of rituximab with Z3 protein variants.

Components	T_m1_ (°C)	T_m2_ (°C)	T_m3_ (°C)	Δ_u_G^0^ kJ mol^−1^	Δ_u_S^0^ kJ mol^−1^ K^−1^	Δ_u_H^0^ kJ mol^−1^
Rituximab	68.5 ± 0.1	74.1 ± 0.0	81.3 ± 0.5	55.8 ± 0.5	1.3 ± 0.0	429.9 ± 1.6
Rituximab: linear Z3 (1:1)	73.4 ± 0.1	84.6 ± 0.1	N/A	68.7 ± 3.3	1.4 ± 0.1	481.1 ± 22.6
Rituximab: cyclic Z3 (1:1)	73.4 ± 0.2	82.8 ± 0.1	N/A	66.8 ± 1.0	1.4 ± 0.0	476.0 ± 9.0

The standard error was calculated from three independent measurements.

## Data Availability

The data supporting this study’s findings are available from the corresponding author upon reasonable request.

## Supplementary Materials

The following supporting information can be downloaded at: https://www.mdpi.com/article/10.3390/ijms24021281/s1.
