# Supplementary material for: Expression and Characterization of Intein-Cyclized Trimer of Staphylococcus aureus Protein A Domain Z
Source: Int J Mol Sci. 2023 Jan 9;24(2):1281. doi: 10.3390/ijms24021281 (PMC9865183; doi:10.3390/ijms24021281)
Supplement: Supplementary file 1 [file ijms-24-01281-s001.zip › ijms-2102791-supplementary.pdf]

Expression and Characterization of Intein-Cyclized Trimer of *Staphylococcus aureus* Protein A Domain Z

Suman Nandy [1,†], Vijay M. Maranholkar [2,†], Mary Crum [1,‡], Katherine Wasden [1,§], Ujwal Patil [2,‖], Atul Goyal [1,¶], Binh Vu [1], Katerina Kourentzi [1], William Mo [1,], Amy Henrickson [3], Borries Demeler [3,4], Mehmet Sen [2,*] and Richard C. Willson [1,2,5,*]**

[1] William A. Brookshire Department of Chemical and Biomolecular Engineering, University of Houston, Houston, TX 77204, USA; suman.che.buet@gmail.com (S.N.); maryancrum@gmail.com (M.C.); kbwasden@gmail.com (K.W.); atulgoyal515@gmail.com (A.G.); vubinh@gmail.com (B.V.); kkourentzi@gmail.com (K.K.); wlmtmsm2@gmail.com (W.M.)

[2] Department of Biology and Biochemistry, University of Houston, Houston, TX 77004, USA; vmaranholkar@uh.edu (V.M.); ujwal47@gmail.com (U.P.)

[3] Department of Chemistry and Biochemistry, University of Lethbridge, Lethbridge, AB T1K 3M4, Canada; amy.henrickson@uleth.ca (A.H.); demeler@gmail.com (B.D.)

[4] Department of Chemistry and Biochemistry, University of Montana, Missoula, MT 59812, USA

[5] Escuela de Medicina y Ciencias de Salud, Tecnológico de Monterrey, Monterrey 64849, Nuevo León, Mexico

* Correspondence: msen2@cougarnet.uh.edu (M.S.); willson@uh.edu (R.C.W.)

† These authors contributed equally to this work.

‡ Current affiliation: Department of Molecular and Human Genetics, Baylor College of Medicine, Houston, TX 77030, USA.

§ Current affiliation: Harvard Medical School, Boston, MA 02115, USA.

‖ Current affiliation: Bioprocess Technologies and Engineering, AstraZeneca, Gaithersburg, MD 20878, USA.

¶ Current affiliation: Vaccine Research and Development, Pfizer, Pearl River, New York, NY 10965, USA.

** Current affiliation: Department of Biomedical Engineering, The University of Texas at Austin, Austin, TX 78712, USA.

S1. Plasmid schematics

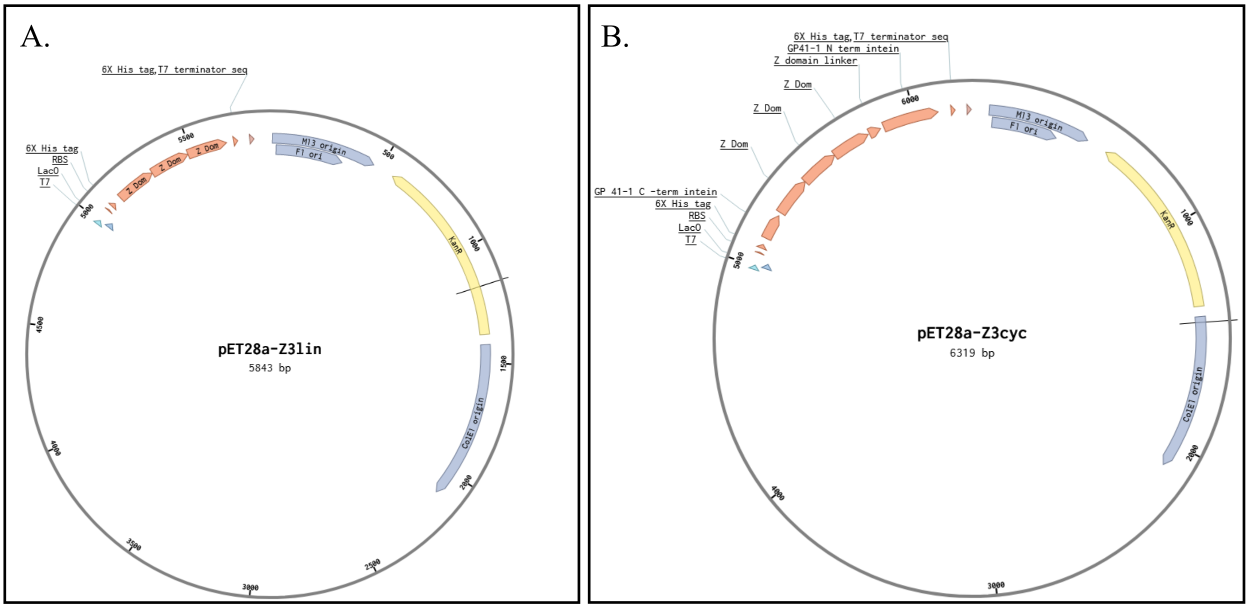

Figure S1: Plasmid schematics. (A) Schematic of pET28a-Z3lin encoding Z3 domain gene. (B) Schematic of pET28a-Z3cyc encoding SICLOPPS construction of the cyclic Z3 domain gene. Drawn on Benchling.com.

S2. Gene sequence

Linear Z3:

The DNA sequences of the gene fragment flanked by the NdeI site (labeled in red) and XhoI site (labeled in blue):

CATATGGTCGATAACAAATTCAACAAAGAACAACAAAACGCGTTCTATGAGATCTTACATTTAC
CTAACTTAAACGAAGAACAACGAAACGCCTTCATCCAAAGTTTAAAAGATGACCCAAGCCAAAG
CGCTAACCTTTTAGCAGAAGCTAAAAAGCTAAATGATGCTCAAGCACCGAAAGTCGATAACAAA
TTCAACAAAGAACAACAAAACGCGTTCTATGAGATCTTACATTTACCTAACTTAAACGAAGAAC
AACGAAACGCCTTCATCCAAAGTTTAAAAGATGACCCAAGCCAAAGCGCTAACCTTTTAGCAGA
AGCTAAAAAGCTAAATGATGCTCAAGCACCGAAAGTCGATAACAAATTCAACAAAGAACAACAA
AACGCGTTCTATGAGATCTTACATTTACCTAACTTAAACGAAGAACAACGAAACGCCTTCATCC
AAAGTTTAAAAGATGACCCAAGCCAAAGCGCTAACCTTTTAGCAGAAGCTAAAAAGCTAAATGA
TGCTCAAGCACCGAAAGTCGACTGATAAGCTTGCGGCCGCACTCGAG

Cyclic Z3 parent gene DNA sequence, including inteins and exteins:

The DNA sequences of the gene fragment flanked by the NdeI site (labeled in red) and XhoI site (labeled in blue):

CATATGATGTTGAAGAAGATCCTGAAGATTGAAGAGTTGGATGAACGCGAACTTATTGATATTG
AAGTCAGCGGGAATCATTTGTTTTATGCTAATGATATTTTGACTCATAACTCTAGTTCcTCGGA
CGTGGTAGACAATAAGTTCAATAAGGAGCAACAGAACGCATTCTATGAAATTCTTCACTTGCCC
AACTTAAACGAGGAGCAACGTAACGCATTTATTCAGTCTTTAAAAGATGATCCTAGCCAATCCG
CCAACTTATTAGCGGAAGCCAAGAAGTTGAATGATGCTCAAGCCCCAAAGGTCGATAACAAATT
CAACAAAGAACAACAAAACGCGTTCTATGAGATCTTACATTTACCTAACTTAAACGAAGAACAA
CGAAACGCCTTCATCCAAAGTTTAAAAGATGACCCAAGCCAAAGCGCTAACCTTTTAGCAGAAG
CTAAAAAGCTAAATGATGCTCAAGCACCGAAAGTCGATAACAAATTCAACAAAGAACAACAAAA
CGCGTTCTATGAGATCTTACATTTACCTAACTTAAACGAAGAACAACGAAACGCCTTCATCCAA
AGTTTAAAAGATGACCCAAGCCAAAGCGCTAACCTTTTAGCAGAAGCTAAAAAGCTAAATGATG
CTCAAGCACCGAAAGTCGATGGCGGGGCAGAGGCTGCGGCTAAGGAAGCAGCCGCTAAAGCAGC
GCCTGTCGACACCCGCAGCGGTTATTGTTTAGACCTTAAGACACAGGTGCAGACTCCACAAGGG
ATGAAGGAAATTTCAAACATCCAAGTAGGCGATCTTGTTCTTTCAAACACCGGGTACAATGAAG
TCCTGAATGTTTTCCCTAAGTCTAAAAAAAAAAGCTACAAAATCACTCTTGAGGACGGAAAAGA
GATTATTTGCAGCGAAGAGCACCTGTTCCCCACGCAAACTGGAGAAATGAATATCAGCGGCGGA
CTTAAAGAGGGTATGTGTTTGTATGTTAAAGAGGGTGGCAGTGACTACAAAGACGATGACGACA
AGTAACTCGAG

S3 Protein Sequence

Cyclic Z3 protein parent gene including inteins and exteins

Thrombin site

His tag

C-intein

```
MGSSHHHHHHSSGLVPRGSHMMLKKILKIEELDERELIDIEVSGNHLFYANDILTHNSSSSDVV
DNKFNKEQQNAFYEILHLPNLNEEQRNAFIQSLKDDPSQSANLLAEAKKLNDAQAPKVDNKFNK
EQQNAFYEILHLPNLNEEQRNAFIQSLKDDPSQSANLLAEAKKLNDAQAPKVDNKFNKEQQNAF
YEILHLPNLNEEQRNAFIQSLKDDPSQSANLLAEAKKLNDAQAPKVDGGAEAAAKEAAAKAAPV
DTRSGYCLDLKTQVQTPQGMKEISNIQVGDLVLSNTGYNEVLNVFPKSKKKSYKITLEDGKEII
CSEEHLFPTQTGEMNISGGLKEGMCLYVKEGGSDYKDDDDK
```

N-intein

Cyclic Z3 protein after SICLOPPS:

C-extein

```
SSSDVVDNKFNKEQQNAFYEILHLPNLNEEQRNAFIQSLKDDPSQSANLLAEAKKLNDAQAPKV
DNKFNKEQQNAFYEILHLPNLNEEQRNAFIQSLKDDPSQSANLLAEAKKLNDAQAPKVDNKFNK
EQQNAFYEILHLPNLNEEQRNAFIQSLKDDPSQSANLLAEAKKLNDAQAPKVDGGAEAAAKEAA
AKAAPVDTRSGY
```

N-extein

Linker

Predicted properties from ExPasy ProtParam tool (https://web.expasy.org/protparam/)
Extinction coefficient: 5,960 $M^{-1}cm^{-1}$
Abs 0.1% (1 g/L): 0.26
Number of amino acids: 204
Molecular weight: 22,719 Da

Linear Z3 protein

His tag　　Thrombin site

MGSSHHHHHHSSGLVPRGSHMVDNKFNKEQQNAFYEILHLPNLNEEQRNAFIQSLKDDPSQSAN
LLAEAKKLNDAQAPKVDNKFNKEQQNAFYEILHLPNLNEEQRNAFIQSLKDDPSQSANLLAEAK
KLNDAQAPKVDNKFNKEQQNAFYEILHLPNLNEEQRNAFIQSLKDDPSQSANLLAEAKKLNDAQ
APKVD

Extinction coefficient: 4,470 $M^{-1}cm^{-1}$
Abs 0.1% (1 g/L): 0.20
Molecular Weight: 22,394 Da

S4 Chromatograms

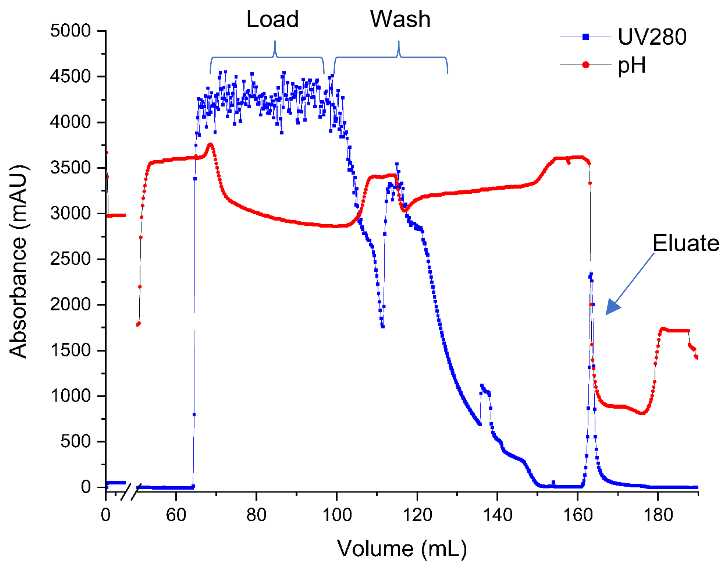

Figure S2: IgG Sepharose affinity chromatographic purification of linear Z3 at a linear flow rate of 30 cm/h (1 mL/min), on an XK 16/20 column packed with 10 mL IgG Sepharose 6 Fast Flow.

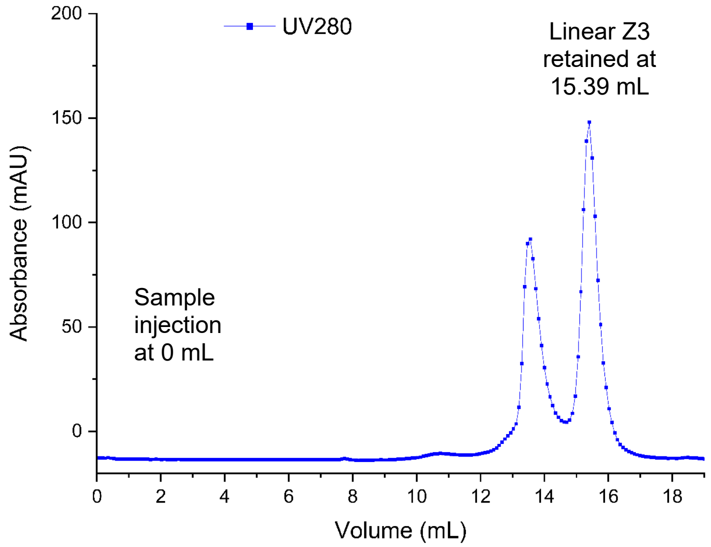

Figure S3: Superdex 200 Increase 10/300 GL SEC column (24 mL bed volume) purification and buffer exchange of linear Z3 at 0.5 mL/min. The retention volume range corresponding to the protein fractionation range (669 kDa to 13.7 kDa) was reported to be 7 mL - 19 mL at 0.5 mL/min.

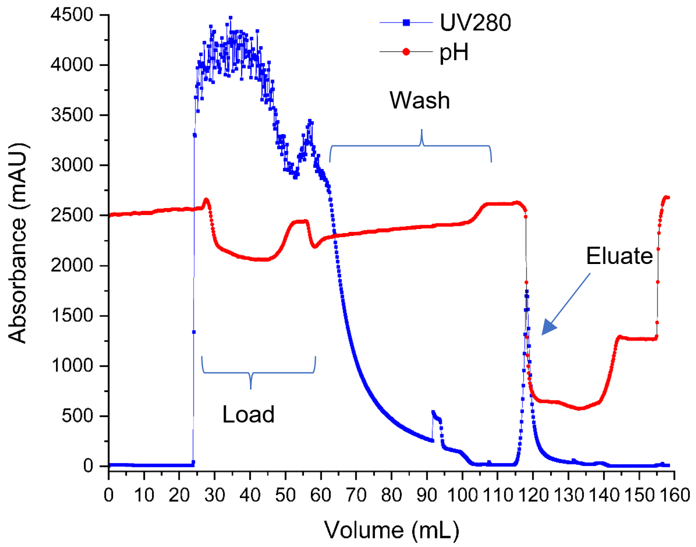

Figure S4: IgG Sepharose affinity chromatography for purification of cyclic Z3. The purification process was performed at a linear flow rate of 30 cm/h (1 mL/min), on an XK 16/20 column packed with 10 mL IgG Sepharose 6 Fast Flow.

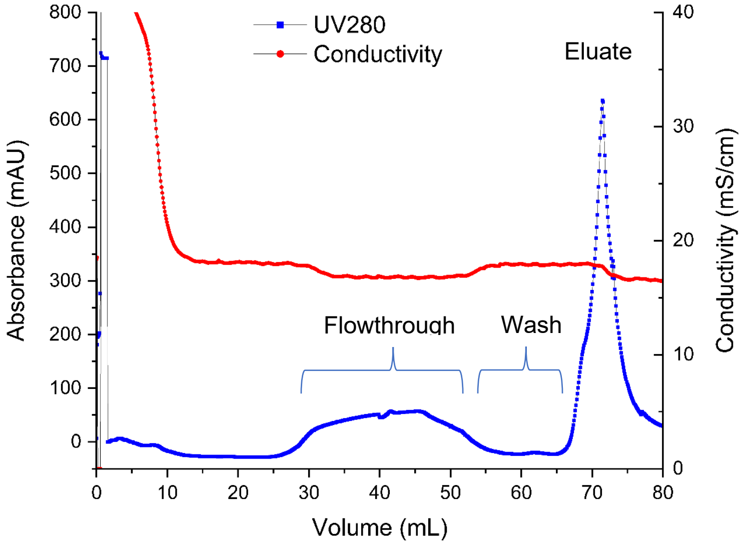

Figure S5: Ni NTA affinity chromatographic separation of cyclized protein from non-cyclized parent protein at a linear flow rate of 30 cm/h (1 mL/min), on an XK 16/20 column packed with 5 mL Ni Sepharose 6 Fast Flow.

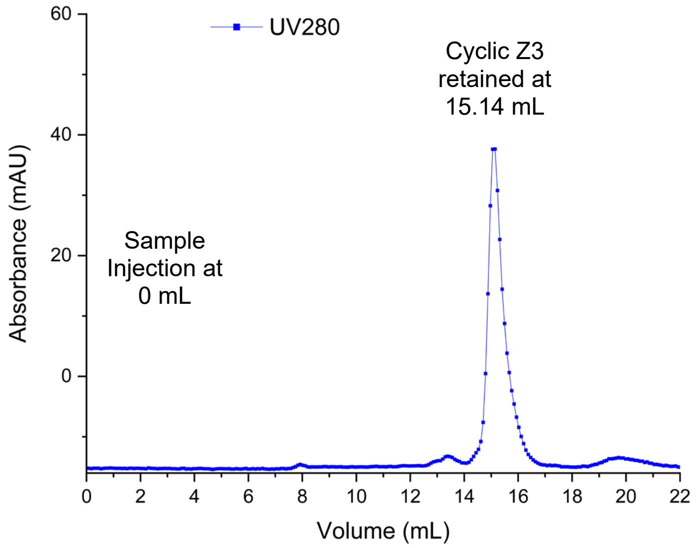

Figure S6: Superdex 200 Increase 10/300 GL SEC (24 mL bed volume) purification and buffer exchange of linear Z3 at 0.5 mL/min. The retention volume range corresponding to the protein fractionation range (669 kDa to 13.7 kDa) was reported to be 7 mL - 19 mL at 0.5 mL/min.

Table S1: Tryptic peptides of linear Z3 detected by timsTOF pro mass spectrometry

Peptide no.	Sequence	Observed mass	Expected mass	Charge (Z)
1	FNKEQQNAFYEILHLPNLNEEQR	2873.41	2873.39	4
2	EQQNAFYEILHLPNLNEEQR	2484.21	2485.69	2
3	NAFIQSLKDDPSQSANLLAEAK	2359.21	2359.19	3
4	GSSHHHHHHSSGLVPR	1767.84	1768.87	3
5	DDPSQSANLLAEAKK	1585.79	1585.78	2
6	DDPSQSANLLAEAK	1457.69	1458.54	2
7	LNDAQAPKVDNK	1311.68	1311.67	2
8	GSHMVDNKFNK	1275.61	1275.59	2
9	LNDAQAPKVD	1069.54	1069.54	2
10	KLNDAQAPK	983.54	983.53	2
11	NAFIQSLK	919.51	920.08	1

Table S2: Tryptic peptides of cyclic Z3 detected by timsTOF pro mass spectrometry

Peptide no.	Sequence	Observed mass	Expected mass	Charge (Z)
1	FNKEQQNAFYEILHLPNLNEEQR	2873.41	2873.39	4
2	EQQNAFYEILHLPNLNEEQR	2484.21	2485.69	2
3	NAFIQSLKDDPSQSANLLAEAK	2359.21	2359.19	3
4	DDPSQSANLLAEAKK	1585.79	1585.78	2
5	DDPSQSANLLAEAK	1457.69	1458.71	2
6	LNDAQAPKVDNK	1311.68	1311.67	2
7	SGYSSSDVVDNK	1256.55	1256.54	2
8	EAAAKAAPVDTR	1198.63	1198.62	2
9	KLNDAQAPK	983.54	983.53	2
10	NAFIQSLK	919.51	920.08	1
11	VDGGAEAAAK	887.43	888.44	2

S5 ITC system suitability test:

A system suitability test was performed by studying the binding of $CaCl_2$ to EDTA, as per the manufacturer's recommendation. A stirring speed of 750 rpm, a temperature of 25 °C, a reference power of 5 µcal/s, high feedback, and an initial delay of 60 s was used for all experiments. 100 µM EDTA in the cell was titrated against 1 mM $CaCl_2$ in the syringe. The first priming injection was 0.4 µL, followed by 16 injections of 2.0 µL with 150 seconds between injections. Figure S8 and figure S9 show the corresponding thermogram and isotherm plots.

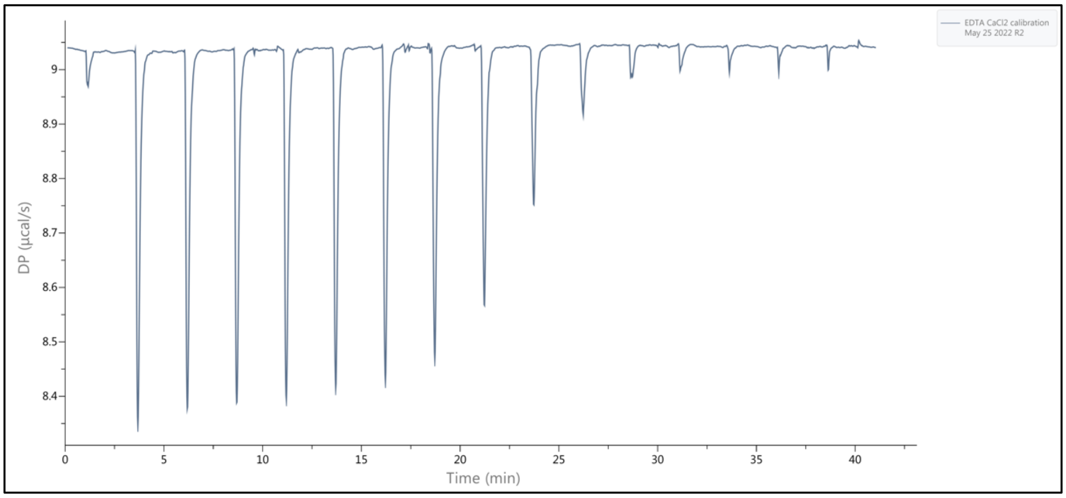

Figure S7: Thermogram of EDTA/$CaCl_2$ titration.

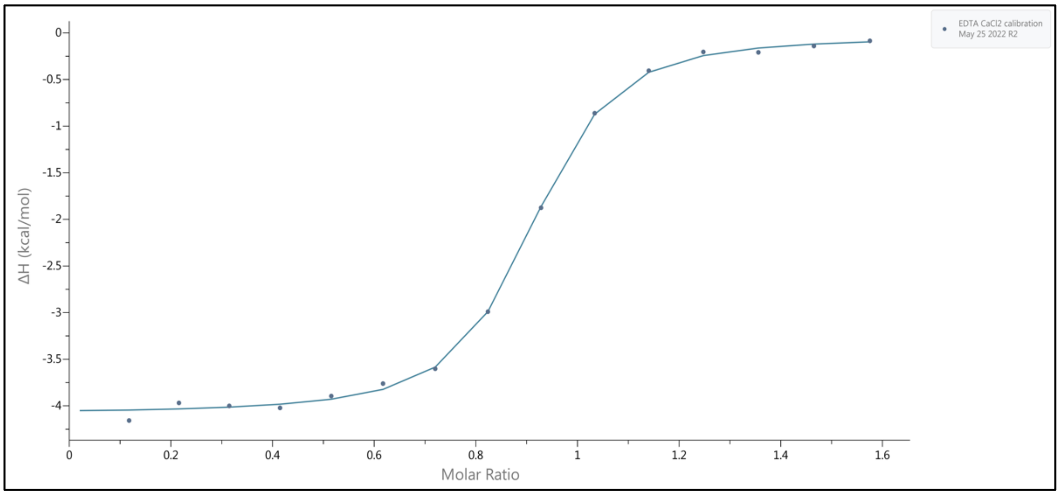

Figure S8: Isotherm plot of EDTA/$CaCl_2$ titration.

Table S3: Thermodynamic parameters of EDTA/$CaCl_2$ titration

	Temp. (°C)	[$CaCl_2$] (mM)	[EDTA] (μM)	K_D (M^{-1})	ΔG (kcal/mol)	ΔH (kcal/mol)	TΔS (kcal/mol)	N (EDTA/$CaCl_2$)
In-house instrument data	25	1	100	698×10^{-9}	-8.4	-4.04	-4.36	0.86
Standard data from the supplier	25	1	100	674×10^{-9}	-8.4	-3.97	-4.44	0.99
